# Visual Acuity Assessment in Children With Autism Spectrum Disorder Using a Child-Friendly Minimum-Separable Chart: A Five-Case Report

**DOI:** 10.7759/cureus.94705

**Published:** 2025-10-16

**Authors:** Yo Iwata, Yuki Kusayanagi

**Affiliations:** 1 Department of Ophthalmology, School of Allied Health Sciences, Kitasato University, Sagamihara, JPN; 2 Department of Ophthalmology Examination, Sakoh Eye Clinic, Kawasaki, JPN

**Keywords:** autism spectrum disorder, child-friendly minimum-separable chart, cooperation rate, pediatric ophthalmology, visual acuity testing

## Abstract

Children with autism spectrum disorder (ASD) often have eye disorders, such as strabismus and amblyopia; thus, it is important to evaluate their visual function. However, achieving their cooperation for visual acuity testing is challenging. We developed a child-friendly minimum separable chart (CFMS chart) using meaningful pictures and two-choice answers. In this study, visual acuity tests were performed using both Landolt rings and CFMS charts in five children with ASD. For the Landolt ring test, only one patient was cooperative, whereas for the CFMS chart, all the patients were cooperative for evaluating the visual acuity of at least one eye. The meaningful visual targets and the two-choice format promoted understanding and interest among children with ASD, making it easier to obtain their cooperation for visual acuity testing. This is the first study to demonstrate the potential of the CFMS chart as a method for assessing visual acuity in children with ASD.

## Introduction

Autism spectrum disorder (ASD) is a neurodevelopmental disorder characterized by a wide range of symptoms, including social communication disorders, limited and repetitive behavior, and sensory hypersensitivity or hyposensitivity [1]. The prevalence of ASD is increasing and was reported to be 2.76% in 2020 [2]. The main factors contributing to this increase include greater awareness, improved diagnostic criteria, and improved detection capabilities; 40%, 20%, 21%, and 10% of the children with ASD reportedly have ophthalmic disorders, severe refractive errors, strabismus, and amblyopia, respectively [3]. Compared to healthy children, children with ASD are two to three times more likely to have strabismus [4-8] and 1.7 times more likely to have amblyopia [4,5,7]. Therefore, ophthalmological examinations are extremely important in children with ASD, and visual acuity testing is the most important test for assessing visual function. Despite this, ophthalmological examinations are often not performed in children with ASD, and hospitals with ASD specialty centers have not conducted or requested ophthalmological examinations in 43% of the cases, which is presumably attributed to the difficulty in conducting such examinations in children with ASD [9].

Subjective responses are generally necessary for evaluating vision. In standard clinical practice, visual acuity is estimated by the smallest resolvable detail (“minimum separable”). Charts such as the Landolt C (Landolt ring) and the Tumbling E implement this principle by requiring the child to judge the orientation of a small gap or arm among four alternatives (up, down, left, right), a task that involves symbolic matching and direction reporting. These cognitive demands can reduce cooperation in many children with ASD. It is particularly important to use an easy-to-understand method of visual acuity testing for children with ASD. We previously developed a child-friendly minimum separable chart (CFMS chart) that enables convenient and accurate measurement of visual acuity in children [10]. The CFMS chart displayed pictures familiar to the children on the left and right sides, with one picture having a notch and the other without.

Children were subsequently asked to identify which of the two pictures had a notch, and their visual acuity was measured. For example, there are pictures of apples on the left and right sides; the apple on the left appears to be bitten, whereas the apple on the right appears to be intact. The patients are asked, “Which apple was eaten?” In the CFMS design, the embedded notch is metrically equivalent to the Landolt gap (minimum separable), preserving the same measurement principle while presenting a meaningful pictorial target. The response is reduced to two alternatives and can be given by simple pointing, thereby lowering cognitive load and improving engagement. Similarly, it is possible to create various types of visual targets and obtain the same visual acuity regardless of the image used [10]. In addition, because two choices are possible, it is easier to comprehend than the four-choice Landolt ring visual acuity chart; thus, more active cooperation can be achieved.

To date, no reports have evaluated visual acuity in children with ASD using CFMS charts. In this study, we report the effective visual acuity testing in five children with ASD using the CFMS chart.

## Case presentation

**Figure 1:**
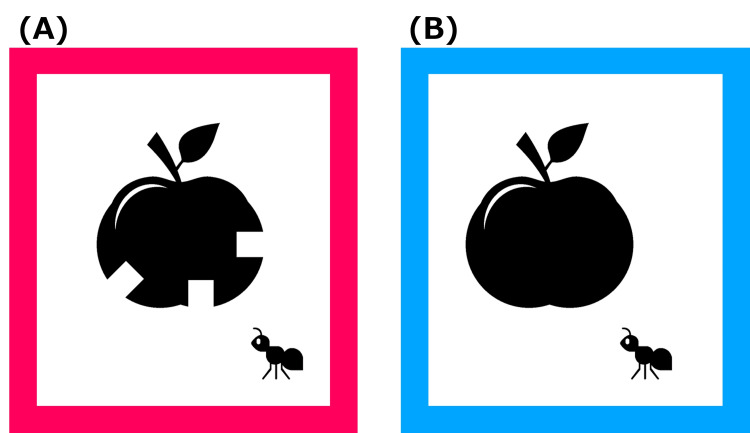
Child-friendly minimum separable (CFMS) chart apple target (A) Apple with a notch; (B) Apple without a notch. The notch in (A) implements the minimum-separable (Landolt C gap) principle; children are asked, ‘Which apple was eaten?’ Image created by Yo Iwata.

**Figure 2:**
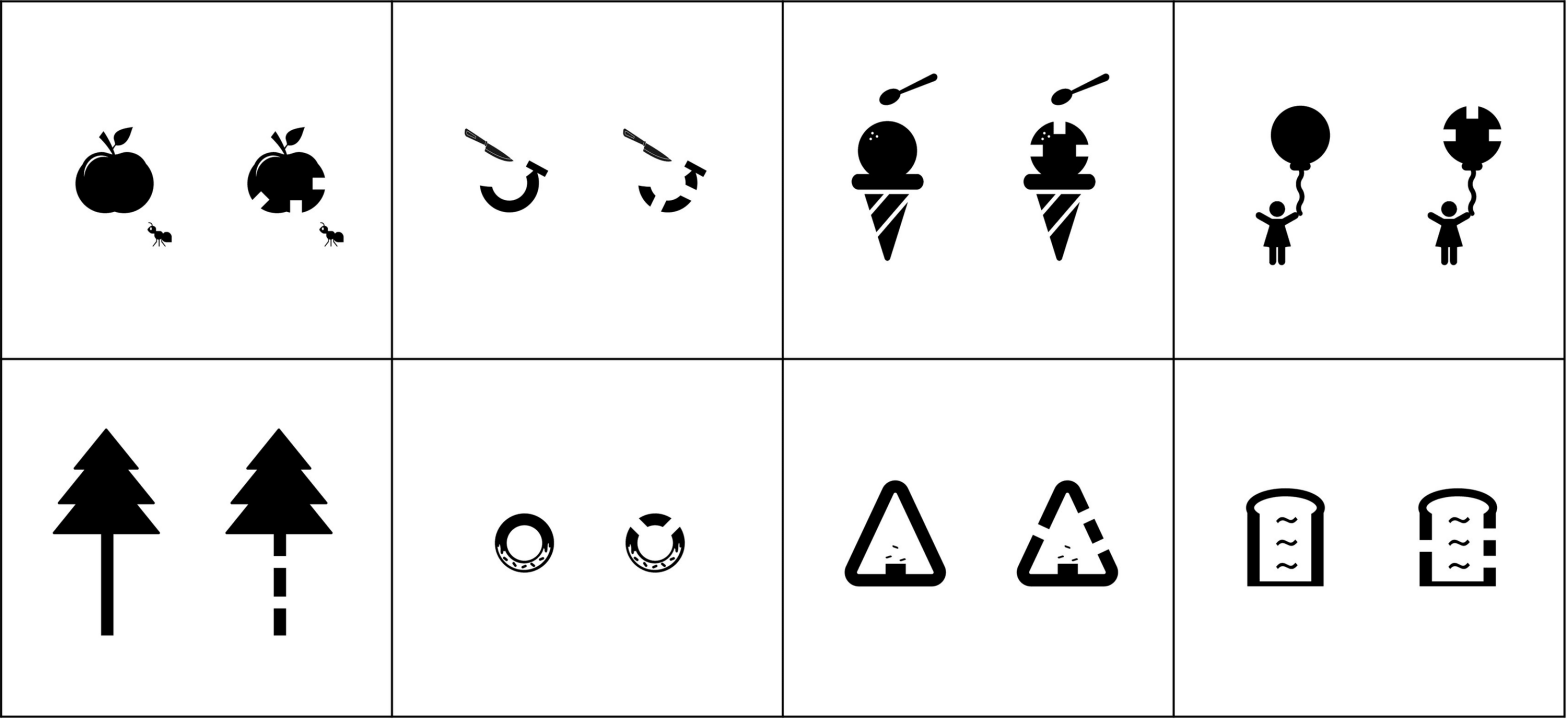
Eight types of targets in the child-friendly minimum separable (CFMS) chart In testing, two copies of the same picture are presented; one includes a notch and the other does not. Because acuity is determined by the notch size (minimum separable), the same visual acuity is obtained regardless of the target selected. Image created by Yo Iwata.

The optotypes used in the CFMS chart are illustrated in Figure 1. In this example, the apple on the left contains a notch and appears to have been eaten, whereas the apple on the right lacks a notch and appears uneaten. A range of optotypes is available in the CFMS charts, as demonstrated in Figure 2.

Visual acuity tests using Landolt rings and CFMS charts were performed in five children with ASD. Visual acuity was assessed using the Landolt ring test in accordance with Japanese Industrial Standards, with a score of three or more out of five correct answers [11]. Each trial has four possible orientations, so a random guess is correct one-quarter of the time and incorrect three-quarters of the time. Meeting the three-of-five criterion by chance requires exactly three, four, or five correct responses. There are 10, five, and one possible arrangements for these outcomes, respectively. Summing their contributions gives:

$$ 10\left(\frac{1}{4}\right)^{3}\left(\frac{3}{4}\right)^{2} + 5\left(\frac{1}{4}\right)^{4}\left(\frac{3}{4}\right) + \left(\frac{1}{4}\right)^{5} = 0.103515625 \approx 0.1035\ \text{(}10.4\%\text{)} \tag{1} $$

**Figure 3:**
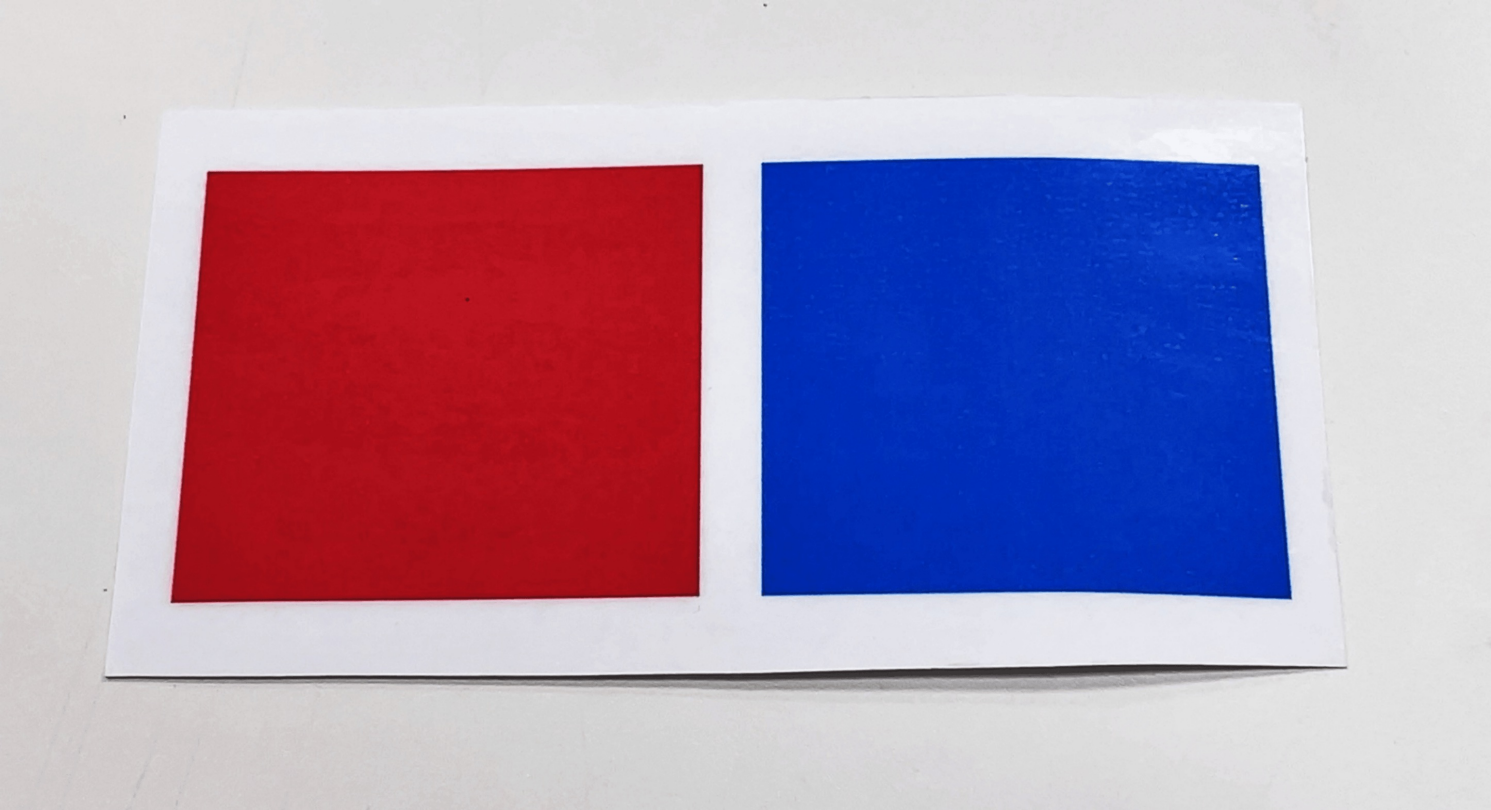
Answer cards used for child-friendly minimum separable (CFMS) charts Children indicated the notch by pointing to a handheld red or blue card that matched the frame color of the picture containing the notch.

The visual acuity test using the CFMS chart asks the patient to indicate which of the two pictures placed side by side has a notch. The image on the left is surrounded by a red frame, and the image on the right is surrounded by a blue frame (Figure 1). Each patient had a card in front of them, with a red square on the left side and a blue square on the right side (similar to an eye chart) (Figure 3). The respondent is asked to point to the red or blue card in their hand to indicate which side of the notch is on. Considering a probability of 10.4% in visual acuity tests using Landolt rings and the number of numbers that could be presented, visual acuity was evaluated as present when three numbers were presented and correctly identified in visual acuity tests using CFMS charts [10].

With two alternatives per item, the probability of three consecutive correct responses is:

$$ \left(\frac{1}{2}\right)^{3} = \frac{1}{8} = 0.125 = 12.5\% \tag{2} $$

Eight types of targets were used in the CFMS chart (Figure 2), and any target that seemed interesting to the child was used.

Table 1 shows the age, sex, ocular deviation, objective refractive, and presence or absence of intellectual disability in five cases of ASD. The children were aged 3-6 years; ID1 had intermittent exotropia, while the other children had slight exophoria. In all cases, no significant refractive abnormalities or anisometropia were observed using an autorefractometer. Children with ID1 and ID3 also had intellectual disabilities, children with ID2 and ID5 did not have intellectual disabilities, and there were no records of intellectual disabilities in children with ID4.

**Table 1:** Information on background factors for all cases Indicates age, sex, ocular deviation, objective reference, and presence or absence of intellectual disability. X(T): intermittent exotropia. X: exophoria.

ID	Age (years)	Sex	Ocular deviation	Objective refraction	Intellectual disability
1	5	Male	F: 25 PD X(T)'	R: S+0.75D C-0.75D Ax 175°	Present
			N: 20 PD X(T)	L: S+0.25D C-0.50D Ax 170°	
2	6	Male	F: 4 PD X'	R: S-1.25D C-0.50D Ax 20°	Absent
			N: 4 PD X	L: S-0.25D C-0.50D Ax 180°	
3	4	Male	F: 2 PD X'	R: S+1.00D C-1.00D Ax 180°	Present
			N: no-shift	L: S+1.25D C-0.25D Ax 165°	
4	3	Female	F: 12 PD X'	R: S+0.50D C-1.00D Ax 180°	Data not available
			N: 8 PD X	L: S+1.00D C-0.75D Ax 10°	
5	6	Male	F: 4 PD X'	R: S-0.75D C-0.50D Ax 175°	Absent
			N: no-shift	L: S-1.00D C-0.25D Ax 160°	

The visual acuity test was first performed using the Landolt ring, followed by an evaluation using the CFMS chart. The test distance was set at 3 m. Uncorrected visual acuity was measured first (right eye followed by the left eye); if it was not 0.0 logMAR, corrected visual acuity was then measured.

The individual cases are described below.

Case 1 (ID1, 5-year-old male). Intermittent exotropia (distance 25 PD; near 20 PD) with intellectual disability. Objective refraction: R S+0.75 D C−0.75 D Ax 175°; L S+0.25 D C−0.50 D Ax 170°. Landolt rings: both eyes uncooperative. CFMS chart: right eye 0.4 logMAR (improving to approximately 0.3 with trial correction near S+0.75 D C−0.75 D Ax 170°); left eye uncooperative.

Case 2 (ID2, 6-year-old male). Mild exophoria (distance 4 PD; near 4 PD) without intellectual disability. Objective refraction: R S−1.25 D C−0.50 D Ax 20°; L S−0.25 D C−0.50 D Ax 180°. Landolt rings: both eyes uncooperative. CFMS chart: both eyes 0.2 logMAR (no correction).

Case 3 (ID3, 4-year-old male). Mild exophoria at distance (2 PD) and orthophoria at near (no-shift), with intellectual disability. Objective refraction: R S+1.00 D C−1.00 D Ax 180°; L S+1.25 D C−0.25 D Ax 165°. Landolt rings: both eyes uncooperative. CFMS chart: right eye 0.3 logMAR; left eye uncooperative.

Case 4 (ID4, 3-year-old female). Mild exophoria (distance 12 PD; near 8 PD); intellectual disability status not recorded. Objective refraction: R S+0.50 D C−1.00 D Ax 180°; L S+1.00 D C−0.75 D Ax 10°. Landolt rings: both eyes uncooperative. CFMS chart: right eye 0.2 logMAR (improving to 0.1 with S+0.50 D C−1.00 D Ax 180°); left eye 0.2 logMAR (no correction).

Case 5 (ID5, 6-year-old male). Mild exophoria at distance (4 PD) and orthophoria at near (no-shift) without intellectual disability. Objective refraction: R S−0.75 D C−0.50 D Ax 175°; L S−1.00 D C−0.25 D Ax 160°. Landolt rings: right eye 0.2 logMAR (no correction); left eye 0.0 logMAR (no correction). CFMS chart: right eye 0.1 logMAR (improving to 0.0 with approximately −0.50 D); left eye 0.0 logMAR.

Table 2 presents the visual acuity (logMAR value) in the five cases.

**Table 2:** Visual acuity (logMAR value) in all cases using Landolt rings and child-friendly minimum separable (CFMS) charts In the Landolt ring test, four out of five cases were uncooperative, whereas in the CFMS chart, all patients demonstrated cooperation for visual acuity testing in at least one eye.

ID	Visual acuity with Landolt ring	Visual acuity with CFMS chart
1	R: Uncooperative	R: 0.4 (0.3×S+0.75D=C-0.75D Ax170)
	L: Uncooperative	L: Uncooperative
2	R: Uncooperative	R: 0.2 (n.c.)
	L: Uncooperative	L: 0.2 (n.c.)
3	R: Uncooperative	R: 0.3
	L: Uncooperative	L: Uncooperative
4	R: Uncooperative	R: 0.2 (0.1×S+0.50D=C-1.00D Ax180)
	L: Uncooperative	L: 0.2 (n.c.)
5	R: 0.2 (n.c.)	R: 0.1 (0×-0.50)
	L: 0	L: 0

**Figure 4:**
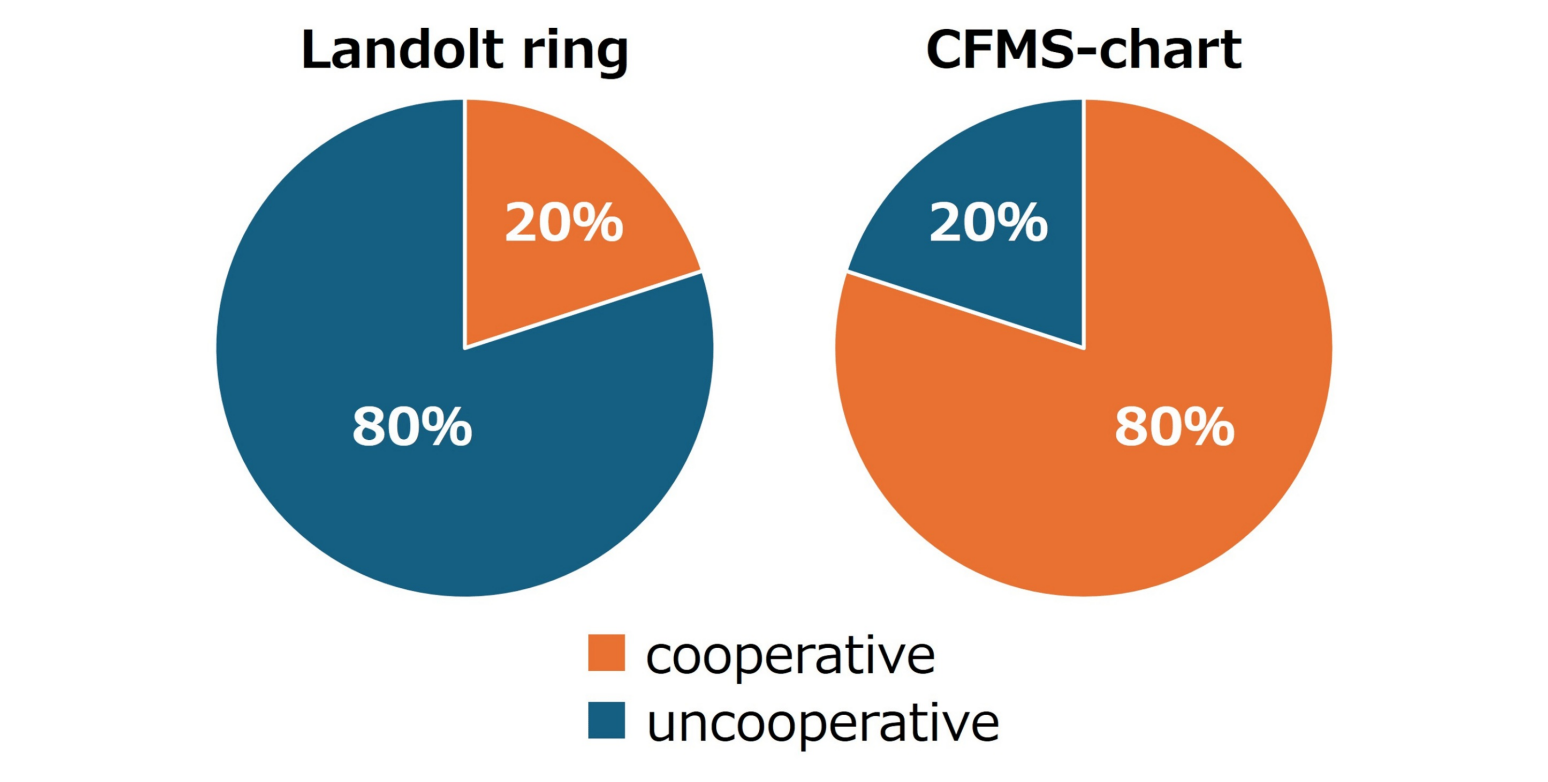
Success rates of visual acuity measurement with Landolt rings and the child-friendly minimum separable (CFMS) chart With Landolt rings, visual acuity measurement succeeded in 2 of 10 eyes; with the CFMS-chart, it succeeded in 8 of 10 eyes.

Across the series, four of five children were unable to complete Landolt C testing, whereas all five cooperated with the CFMS chart in at least one eye (three children in both eyes: ID2, ID4, ID5). At the eye level, visual acuity was measurable in two of 10 eyes (20%) with Landolt C versus eight of 10 eyes (80%) with the CFMS chart; the remaining eyes were uncooperative (Figure 4). The range of visual acuity measured with the Landolt ring and the CFMS chart was 0.4-0.0 logMAR.

This report focuses on the feasibility of distance visual acuity testing in children with ASD within an ophthalmic clinical setting. Detailed neuropsychiatric profiling or systemic comorbidity work-up was outside the scope of this study and was not collected.

## Discussion

In this study, four out of five patients were unable to cooperate in the visual acuity test using the Landolt ring; however, all patients were able to cooperate for visual acuity testing of at least one eye using the CFMS chart, suggesting that understanding and participation were possible with the CFMS chart. Although the CFMS chart was administered after the Landolt ring test and the two tests differ markedly in method, a practice effect cannot be completely excluded. Additionally, with the CFMS chart, two cases were uncooperative only in the left eye, which may reflect waning attention or fatigue as testing progressed.

A study investigating the cooperation rate for vision screening in children with ASD reported a cooperation rate of 33% for the Tumbling E chart, 50% for Lea symbols, and 60% for pediatric picture-colored visual acuity charts [12]. These findings align with those of our study, suggesting that the comprehension of abstract task formats, such as the Tumbling E chart and Landolt rings, which require responding to the direction of the notches, by children with ASD is challenging. Tumbling E and Landolt rings are abstract shapes with little meaning and are used only in vision tests. Children with ASD are more likely to learn, understand, and participate in tests when pictures and symbols with meaning are used rather than shapes [13,14]. The low cooperation rate of children with ASD is related to whether figures are abstract and whether they have meaning.

In the CFMS chart employed in this study, meaningful pictures were used as visual cues, which presumably promoted sustained interest and ease of understanding among children with ASD as well as active participation in the test, resulting in a high cooperation rate. The advantages of using the CFMS chart for children with ASD include not only the use of meaningful pictures as visual cues, but also the two-choice question format (“Which one?”). The Landolt ring chart has a choice of four options (up, down, left, or right) that may be difficult for children with ASD to understand and interpret. However, in the CFMS chart, the choice is made between two options, which makes it easier. Past studies have also revealed that, in stereoscopic vision tests targeting 3-year-olds, two-choice tasks demonstrated higher test cooperation rates than four-choice tasks, indicating that fewer choices are easier to understand and cooperate with [15]. 

Taken together, these results imply that chart design features - semantically meaningful pictures and a binary “Which one?” response mapping - were effective in sustaining attention and clarifying the task for children with ASD, thereby increasing cooperation.

As childhood is a very important period for the development of visual function, the American Academy of Ophthalmology and the American Association for Pediatric Ophthalmology and Strabismus recommend that children undergo annual vision screening until the age of 3-5 years [16]. Children with ASD are at a higher risk of developing eye diseases than children without ASD [3]; therefore, it is particularly important for them to undergo annual vision screening. The Landolt ring, which has been specified by the International Organization for Standardization as a measure of visual acuity [17], is reportedly associated with low cooperation rates in children [18,19]; moreover, children with ASD have even lower cooperation rates. Therefore, a simpler and easier-to-understand visual acuity test method, such as the CFMS chart, is warranted.

As a feasibility series conducted in an ophthalmic clinic, this study is limited by the small sample size (five cases), single-site design, documentation of intellectual disability as a binary variable without standardized severity metrics, and the absence of detailed neuropsychiatric profiling or systemic comorbidity data, which may limit generalizability. Larger, multi-center studies incorporating standardized ASD measures are warranted.

## Conclusions

In this case report, visual acuity testing with conventional Landolt rings proved challenging for children with ASD, whereas acuity assessment was achievable with the CFMS chart in at least one eye. These preliminary, descriptive findings suggest that the CFMS chart, which uses meaningful pictures and a two-choice format, may be a more feasible and easier-to-understand option that facilitates cooperation.
